# Process evaluation of the data-driven quality improvement in primary care (DQIP) trial: active and less active ingredients of a multi-component complex intervention to reduce high-risk primary care prescribing

**DOI:** 10.1186/s13012-016-0531-2

**Published:** 2017-01-07

**Authors:** Aileen Grant, Tobias Dreischulte, Bruce Guthrie

**Affiliations:** 1 Faculty of Health Sciences and Sport, University of Stirling, Stirling, UK; 2 Medicines Governance Unit, NHS Tayside, Dundee, UK; 3 Population Health Sciences Division, Medical Research Institute, University of Dundee, Dundee, UK

**Keywords:** General practice, Family practice, Prescribing, Quality and safety, Randomised controlled trials, Process evaluation

## Abstract

**Background:**

Two to 4% of emergency hospital admissions are caused by preventable adverse drug events. The estimated costs of such avoidable admissions in England were £530 million in 2015. The data-driven quality improvement in primary care (DQIP) intervention was designed to prompt review of patients vulnerable from currently prescribed non-steroidal anti-inflammatory drugs (NSAIDs) and anti-platelets and was found to be effective at reducing this prescribing. A process evaluation was conducted parallel to the trial, and this paper reports the analysis which aimed to explore response to the intervention delivered to clusters in relation to participants’ perceptions about which intervention elements were active in changing their practice.

**Methods:**

Data generation was by in-depth interview with key staff exploring participant’s perceptions of the intervention components. Analysis was iterative using the framework technique and drawing on normalisation process theory.

**Results:**

All the primary components of the intervention were perceived as active, but at different stages of implementation: financial incentives primarily supported recruitment; education motivated the GPs to initiate implementation; the informatics tool facilitated sustained implementation. Participants perceived the primary components as interdependent. Intervention subcomponents also varied in whether and when they were active. For example, run charts providing feedback of change in prescribing over time were ignored in the informatics tool, but were motivating in some practices in the regular e-mailed newsletter. The high-risk NSAID and anti-platelet prescribing targeted was accepted as important by all interviewees, and this shared understanding was a key wider context underlying intervention effectiveness.

**Conclusions:**

This was a novel use of process evaluation data which examined whether and how the individual intervention components were effective from the perspective of the professionals delivering changed care to patients. These findings are important for reproducibility and roll-out of the intervention.

**Trial registration:**

ClinicalTrials.gov, NCT01425502.

**Electronic supplementary material:**

The online version of this article (doi:10.1186/s13012-016-0531-2) contains supplementary material, which is available to authorized users.

## Background

### High-risk prescribing in primary care

High-risk prescribing in primary care is a major concern for health-care systems internationally. Between 2 and 4% of emergency hospital admissions are caused by preventable adverse drug events [1, 2]. The National Institute of Clinical Excellence (NICE) estimated in 2015 that avoidable drug-related admissions in England cost commissioners £530 million per year [3], and the combined cost of drug-related hospital admissions, emergency department and outpatient visits in the USA was estimated at $19.6 billion in 2013 [4]. A large proportion of these admissions are caused by high-risk prescribing of commonly prescribed drugs, with non-steroidal anti-inflammatory drugs (NSAIDs) and anti-platelets being the main or among the main drugs implicated, causing gastrointestinal, cardiovascular, and renal adverse events [5–7].

### Data-driven quality improvement in primary care (DQIP) intervention and trial

In the UK, virtually, all primary care prescribing is done by general practitioners (GPs). The DQIP intervention was systematically developed and optimised [8–10] and comprised three intervention components: (1) professional education about the risks of NSAIDs and anti-platelets via an educational outreach visit by a pharmacist, written educational material, and regular newsletters which also provided feedback on progress after the practice started the intervention; (2) financial incentives to review patients at the highest risk of NSAID and anti-platelet ADEs, split into a participation fee of £350 and £15 per patient reviewed; and (3) access to a web-based IT tool (which extracted data from GP practice systems to measure practice rates of high-risk prescribing, identify patients for review, bring together relevant data from different parts of the GP record to make review easier, and allowed recording of review decisions to ensure appropriate follow-up), to identify such patients and support structured review. The DQIP intervention was evaluated in a pragmatic cluster randomised controlled stepped wedge trial [9] in 33 practices from one Scottish health board, where all participating practices received the intervention but were randomised to one of ten different start dates [11]. The primary outcome of the trial was a composite of nine NSAID and anti-platelet prescribing indicators. The trial analysis of the primary outcome showed that across all practices, the targeted high-risk prescribing fell during the intervention period (from 3.7% immediately before to 2.2% at the end of the intervention period (adjusted OR 0.63 [95%CI 0.57–0.68], *p* < 0.0001). The intervention only incentivised review of ongoing high-risk prescribing, but led to reductions in both ongoing (1.5% at end vs. 2.6% pre-intervention, adjusted OR 0.60 [95%CI 0.53 to 0.67], *p* < 0.001) and ‘new’ high-risk prescribing (0.7 vs. 1.0%, adjusted OR 0.77 [0.68 to 0.87], *p* < 0.001). Notably, reductions in high-risk prescribing were sustained in the year after financial incentives stopped. In addition, in trial pre-specified secondary analysis, there were reductions in emergency hospital admissions with gastrointestinal ulcer or bleeding (from 55.7 to 37.0/10,000 person-years, RR 0.66 [95%CI 0.51–0.86], *p* = 0.002) and heart failure (from 708 to 513/10,000 person-years, RR 0.73 [95%CI 0.56–0.95], *p* = 0.02) [12].

### Process evaluation of the DQIP intervention

**Fig. 1:**
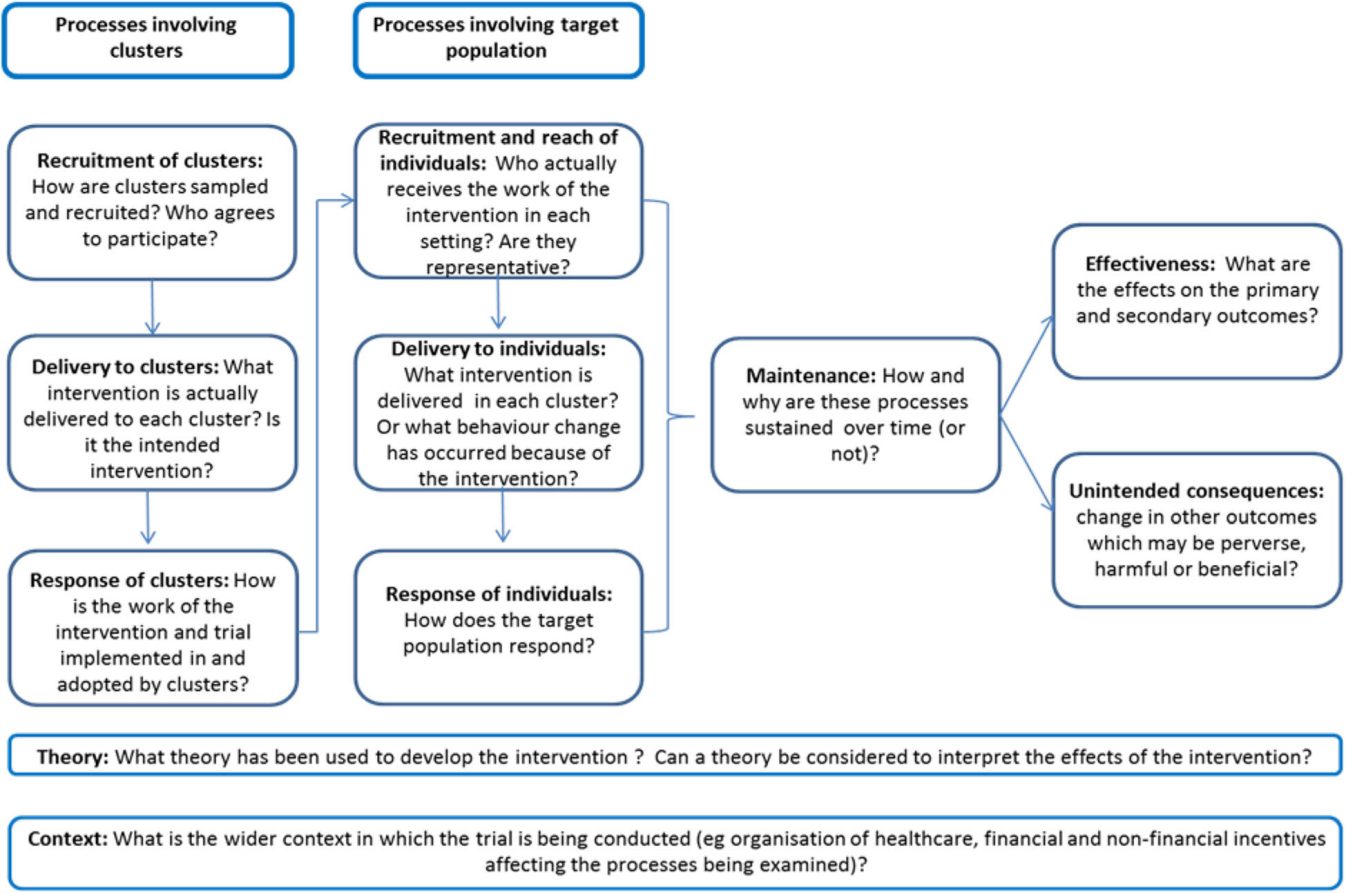
DQIP process evaluation framework. (This paper is reporting column one, the intervention delivered to professionals)

Descriptions of complex interventions in the research literature often lack details about the context in which interventions were delivered and about the delivery and implementation of individual intervention components [13–15]. Such details are however important to decide whether and how an intervention can be implemented in routine care and to inform future research [14, 15]. Alongside the main DQIP trial, we therefore carried out a comprehensive mixed-methods process evaluation [16, 17] based on a cluster-randomised trial process-evaluation framework which we developed [18]. Our framework emphasises the importance of considering two levels of intervention delivery and response that often characterise cluster-randomised trials of behaviour change interventions (although their relative importance will depend on intervention design). The first is the intervention that is delivered to clusters, which respond by adopting (or not) the intervention and integrating it with existing work. The second is the change in care which the cluster professionals deliver to individual patients. In DQIP, the delivery of the intervention to professionals was pre-defined, intended to be delivered with high fidelity across all practices and under the control of the research team, whereas the intervention delivered to patients was largely at the discretion of practices, who decided whether and how they reviewed patients and whether to change prescribing in those reviewed (similar to most health service interventions of this nature). We used this framework to structure our parallel process evaluation, mapping data collection to a logic model of how the data-driven quality improvement in primary care (DQIP) intervention was expected to work (Fig. 1).

Our process evaluation was also informed by normalisation process theory which assists exploration how interventions become integrated, embedded, and routinized into social contexts [19]. Normalisation process theory (NPT) is a theory of implementation designed to assist interpretation of how interventions or new work practices are embedded, enacted, and operationalised within healthcare settings. Interventions or practices become routinely embedded through people working individually or collectively to enact them. This theory is made up of four constructs: *coherence* which refers to participants understanding of the intervention; *cognitive participation* which focuses on enrolment and engagement with the work; *collective action* focuses on how the work was carried out; and *reflexive monitoring* is about how participants assess their progress. NPT had utility qualitatively in sensitising the research team to response in relation to whether and how the intervention was incorporated into practice from the professional’s perspective, and quantitatively in designing measures to assess implementation of the DQIP intervention.

### Focus of this paper

The focus of this paper is on practice participant perceptions of the intervention delivered by the research team to participating practices, which had financial incentive, educational, and informatics components. Complex interventions which have multiple components are common, usually because researchers believe that components will be complementary in terms of being either additive in their effect or synergistic (the whole being greater than the sum of its parts). In the analysis of the main trial, it is not possible to disentangle which components are effective or necessary. The aim of the analysis reported in this paper was therefore to examine professionals’ perceptions of, and responses to the multicomponent intervention delivered to practices, and at which point in recruitment and implementation these components were perceived as more or less active. The study was reviewed by the Fife and Forth Valley Research Ethics Committee (11/AL/0251), and informed consent was obtained from all participants to participate and to publish anonymised data.

## Methods

The overall design and methods have been described previously in the published protocol [20]. In brief, the overall design was a mixed methods parallel process evaluation which examined a set of pre-defined processes and their associations with change in high-risk prescribing at practice level. The quantitative element examined how change in prescribing at practice level was associated with practice characteristics and practice implementation of key processes and is reported separately (﻿Process evaluation of the data-driven quality improvement in primary care (DQIP) trial: quantitative ﻿examination of representativeness of trial participants and heterogeneity of impact. Submitted). The qualitative element consisted of comparative case studies in 10 of the 33 participating practices purposively sampled using maximum variation sampling [21] to include a mix of those initially responding and not responding to the intervention by rapidly reducing their high-risk prescribing, as judged by visual inspection of run charts approximately 4 months after practices started the intervention. The case-study analysis of how practices adopted, implemented, and maintained the intervention is described separately (Process evaluation of the data-driven quality improvement in primary care (DQIP) trial: case-study evaluation of adoption and maintenance of a complex intervention to reduce high-risk primary care prescribing. Submitted). This paper examines professional participant perceptions of the multicomponent intervention delivered to practices using qualitative analysis of interview data collected in the case-study practices.

In each practice, all interviews were carried out by AG (a researcher over 10 years of qualitative experience and already known to two of the practices from a previous project examining prescribing behaviour) [22] with the most involved GP and one other GP, the practice manager and any attached primary care pharmacist approximately 6 months after the practice started the intervention, and the most involved GP again 9 to 12 months after starting the intervention to explore changes over time. Interviews were facilitated by a NPT informed topic guide and lasted approximately 1 h. As part of the intervention in all practices, the AG accompanied the pharmacist (TD) on the educational outreach visit (EOV) and made field notes detailing attendance and the practice’s response. Data was gathered between September 2011 and December 2013 and was in parallel with the trial to capture changes over time.

The analysis was concurrent and iterative with data generation allowing issues and themes identified to inform subsequent data generation and facilitate greater exploration. The analysis continued after data generation until no new themes emerged. The analysis was carried out by AG with BG contributing through discussion of data and interpretation until he became aware of the trial results, and was completed by AG before she knew the outcome of the trial, meaning that qualitative interpretation was blind to trial findings and detailed quantitative process evaluation data. Interview audio-recordings were transcribed verbatim. To preserve anonymity, some identifiable details have been changed and pseudonyms used. A coding frame was developed inductively from field notes and initial interviews and based on our topic guides, framework [18], and logic model [20]. The constant comparative method facilitated revision through detailed analysis [23]. This coding frame was systematically applied to all data, facilitated by NVIVO 8. Analysis utilised the framework technique [24] and NPT as a conceptual framework [19]. AG analysed the data collected in the study twice, inductively letting themes emerge from the data and deductively based on normalisation process theory. NPT interpretation and coding reliability was established through a workshop with NPT experts. In this paper, we drew on analysis from the coherence construct which relates to participant’s perceptions of the intervention which was useful in identifying intervention components from the participant’s perspective. In our accompanying papers, we present the qualitative analysis from the remaining NPT constructs: cognitive participation, collective action, and reflective monitoring (Process evaluation of the data-driven quality improvement in primary care (DQIP) trial: case-study evaluation of adoption and maintenance of a complex intervention to reduce high-risk primary care prescribing. Submitted) and the quantitative data analysis from the NPT informed questionnaires (Process evaluation of the data-driven quality improvement in primary care (DQIP) trial: quantitative examination of representativeness of trial participants and heterogeneity of impact. Submitted). The data was explored for negative cases. Practice names have all been anonymised.

## Results

The findings presented are from 38 professional interviews (ten lead GPs of whom nine were interviewed twice, seven GPs less involved with DQIP, nine practice managers/administrators and three practice pharmacists and from approximately 11 h of field notes from each case study’s EOV (one practice requested and received the education and training twice because of initial implementation failure).

Tables 1 and 2 provide a detailed description of the intervention components defined in the published protocol [15, 20] based on the TIDieR checklist [15] with illustrative study materials provided in the Additional files 1, 2, and 3. The intervention delivered to practices had three main components: (1) financial incentives; (2) education; and (3) a web-based informatics tool which used data extracted from GP electronic medical records. Each of these three main components had a number of subcomponents, included in the intervention for a range of rationales which are detailed in Table 2. Participant’s perceptions of these components and when they were perceived to have an effect are summarised in Table 2 and discussed in detail below.

**Table 1:** TIDieR description of the intervention (item 2 is shown in Table 2)

Intervention component and subcomponents—materials (item 3) and procedures (item 4)	Who provided (item 5), how delivered (item 6), where delivered (item 7), when and how much delivered (item 8), how tailored (item 9)
Financial incentives	
Up-front payment	£350 (547 USD, 497 EUR) paid to every practice paid by the research team immediately before practice started the intervention, the same for every practice.
Payment per review	£15 (23 USD, 21 EUR) per review completed, paid by the research team after the end of the 48-week intervention period once the practice submitted an invoice, the same for every practice.
Education	
Branding intervention ‘patient safety’	Research team used the term ‘patient safety’ in all communications with all practices.
Prescribing advice	Written by the research team for all practices (not tailored) and communicated and distributed at EOV on a one page laminated sheet.
Structured written educational material reinforcing EOV	Written by the research team for all practices (not tailored) and available electronically from the tool and distributed at the EOV.
Educational outreach visit (EOV)	1 hour face-to-face meeting held in the practice and delivered by the research team to a common basic structure, but tailored according to practice interests and expressed needs (Additional file 1). The EOV summarised the latest research evidence, provided clear prescribing advice, and included training on the tool.
Discussion about potential process to do the work	Discussions facilitated by research team during EOV tailored to specific practice characteristics and wishes.
Newsletters	The research team sent a practice progress report to the lead GP and practice manager 8 weekly both before the practice started the intervention (non-tailored update on the progress of the trial and a reminder of the practice start date) and during the intervention period (tailored to reflect practice progress by providing a run chart, commentary on practice progress including comparison to other practices at the same point in implementing the intervention, and offers of further support) (Additional file 2).
Informatics tool	
Identification of patients to review	Web-based tool hosted by NHS Tayside, and the same for every practice. Updated weekly using data-extracted from the general practice’s own clinical records. Practice’s had controlled access through personal log in details via the NHS intranet. At log-in, an updated list of patients needing review was available (Additional file 3: Appendix Figures A1 and A3).
Summarise clinical information to facilitate review	The web-based tool provided a structured summary of relevant patient clinical information to facilitate review (summarised risk factors for relevant adverse drug effects, summary of recent relevant prescribing, Additional file 3: Appendix Figure A4).
Recording of review decisions	
Run charts of change in prescribing	GPs were required to record their review decisions within the informatics tool in order to receive payment (Additional file 3: Appendix Figures A4 and A5. This information was used to ensure that reviewed patients where prescribing was judged appropriately were not repeatedly flagged for review (to increase efficiency by avoiding pointless re-review) except where prescribing judged inappropriate was restarted (to ensure follow-up of patients where intended care was not delivered).
	Timely visual feedback of progress (also used in 8 weekly newsletters in ‘education’) tailored for reach practice and available to view at any time within the informatics tool (Additional file 3: Appendix Figure A2).

**Table 2:** Summary of the active and less active components of the DQIP intervention

Intervention component and subcomponents	Research team’s rationale(s) for including this component (TIDieR item 2)	Participant’s perceptions and/or use of the intervention components
Financial incentives		
In general	Attract practices to participate	Important for recruitment as symbolised recognition of the additional work required of GPs and generated extra income.
Up-front payment	Increase practice commitment to doing the work as already accepted some payment	Had a limited role in mediating effectiveness.
Payment per completed review	Ensure reach is maximised and work is maintained over trial duration	Practices said the financial incentive did not change what they did but two failing practices said had they known about the financial incentive they may have done more.
Education		
Branding DQIP patient safety	Motivate GPs by appealing to their professional values	Important for recruitment as most GPs felt they could not ignore this topic.
Prescribing advice	Avoid inertia	Had an important role in mediating effectiveness because GPs valued clear and concise prescribing advice and were able to action decisions quickly.
Structured written educational material reinforcing EOV	Support and reinforce the educational messages delivered in the EOV	No perceived role in effectiveness. Two GPs used the one page laminated sheet when communicating with patients. Otherwise, this material was not referred to.
Educational outreach visit	Persuade the GPs that the prescribing mattered and encourage GPs to perceive this as new and necessary work which required immediate attention	Had a limited role in mediating effectiveness because already persuaded GPs said they did not find the messages ‘new’, and the already less convinced GPs were not always persuaded that this was a problem worthwhile addressing.
Discussion about potential process to do the work.	Motivate GPs to commence review immediately.	Had an important role in large practices for identifying an appropriate process and defining roles and responsibilities.
Newsletters	Aimed to encourage continued reviewing activity.	Encouraged non-reviewers to revisit tool. Reviewers liked seeing their high risk prescribing going down.
Informatics		
Identification of patients to review	Mobilise GPs to review by reducing administrative burden (at the time of the trial this was a labour intensive process primarily conducted by pharmacists and administrative staff).	Important for implementing change as GPs valued the tool’s simple case finding ability and did not question its accuracy.
Structured clinical information to facilitate review	Facilitate efficient reviews by providing relevant information (reviewing was time consuming as involved reading patient’s notes to identify relevant information).	Important for effectiveness as GPs legitimised and valued the relevant and accurate data; however, all GPs continued to consult patient’s clinical notes.
Record review decisions	Record data important for the trial and process evaluation.	Some GPs found the requirement to ensure all relevant information was addressed irritating.
Run charts of change in prescribing	Motivate GPs to continue reviewing by comparison to previous performance	Had a limited role mediating effectiveness because GPs were not generally motivated by this in the web-based tool, although the same run charts were motivating for some when sent in newsletters.

### Financial incentive

GPs perceived that the offer of a financial incentive was important during recruitment since it was recognition of the additional work to be done in the context of already stretched work schedules and offered a means of generating extra income for the practice. The financial incentive was structured with an upfront payment of £350 ($600, €440) paid after the EOV and £15 ($25, €19) per patient reviewed paid after the end of the intervention in the practice. Most GPs said that the per-patient fee did not actually change whether or not they reviewed patients. However, in some practices, sampled for the case study analysis as early implementation failures (Orosay, Hellisay and Boreray), the interviewed GP felt increased awareness of the financial structure or payment could have facilitated greater implementation of changes in care for patients. In contrast, GPs in some practices which implemented the intervention immediately felt that DQIP was *easy* money for work they should already be doing. One GP went as far as questioning the legitimacy of paying GPs for safety work:

“… whether we should be earning for doing that particular thing I think I would feel a little bit … mmm, you know, this is something that we should be picking up on probably without someone dangling a carrot really …” (Hirta Practice, GP 3 Interview).

Some practices had to be repeatedly reminded to invoice for the work done, supporting the belief that in at least some practices, financial incentives played a limited role in mediating effectiveness even though the offer of payment helped to get initial engagement during recruitment.

### Education

The educational component had several elements; written material summarising the literature and providing prescribing advice, tailored newsletters summarising practice progress and offering support, and an EOV which both targeting knowledge and attitudes, and facilitated discussion of how practices were going to organise to do the DQIP work. All of these and the initial recruitment material were designed so that they clearly branded the work as being about patient safety.

#### Branding as patient safety and the NSAID and anti-platelet topic

Generally, participants in practices in the process evaluation said they signed up to the DQIP trial because they perceived that prescribing safety was clearly about good patient care and was therefore work they should be doing. GPs said that the NSAID and anti-platelet topics covered by DQIP were well-known safety issues, and the logic of the trial resonated with messages they had received from other sources, such as in Health Board organised ‘protected learning time’ educational sessions and from their practice pharmacist. As a result of this shared understanding, they perceived that their practice required good justification for not signing up for an intervention which targeted a well-known patient safety risk and paid them to do so. This high legitimation of the work also facilitated implementation of the intervention in all but four practices which took part in the trial and facilitated expeditious implementation in some practices.

“I felt this was actually genuinely useful, and of benefit to the patients… we’ve always had a bit of an interest in prescribing actually and quite enjoyed some of the little projects that we’ve done with the pharmacist…he is supporting this because it covers some of the issues we’ve already covered but in less detail.” (Monach Practice, GP 1 interview)

### Prescribing advice, structured written educational material and educational outreach visit

The summary of the literature and educational material aimed to improve knowledge and motivate GPs to carry out the reviews. This was valued because it provided clear and up-to-date recommendations, brought the latest evidence and recommendations to the fore of their minds and aided consistent prescribing behaviour.

… it, it was good, there were references that you could go away along and, and read if you wanted … that’s one of the benefits of these things, is if somebody’s done the research and presents it to you and we’re all singing from the same Hymn sheet.” (Hirta Practice, GP 1 interview)

However, these messages were not viewed as ‘new’, and although participants valued the educational material, they did not refer to it while delivering care to patients, perhaps reflecting that its messages were easily internalised. These views were consistent across all participants in the process evaluation. Participants contrasted this summary material with what was normally provided during other improvement activities, which was typically longer, less focused and perceived as being less useful.

Overall, the EOV was viewed by interviewees as useful but not essential (see Additional file 1 for the presentation used). They felt it was useful to have a verbal summary of the literature and recommendations and an overview of the DQIP tool, but these were not felt to be essential to implementing effective reviews. Consistent with this, some GPs who led on delivering their practice’s reviews using the tool did not attend the EOV. Interestingly, one practice (Orosay Practice) which failed to implement the DQIP intervention by not conducting any reviews using the tool still observed a reduction in the targeted prescribing, potentially because of an effect from the educational material.

“Yeah it’s been because of DQIP really yes, aye. Well I suppose we were always anxious prescribing in the elderly… eh, but it’s merely made me sit up and pay a bit more attention…

AG: when you see the patient’s name on the tool or now more generally are you better informed?

It’s more now when I see them, and I didn’t even know if their name’s on the tool …em it’s more of when I see them and I see that they’re on it (NSAID or antiplatelet).”(Orosay Practice, GP 1 interview)

#### Discussion about potential process to do the work

During the EOV, practices were offered the opportunity discuss the ‘best’ process by which they could manage the work load. For some practices, these discussions played an important role in defining how they would organise the work, including roles and responsibilities. These discussions ensured the work commenced shortly after the EOV in the large initial implementation practices where administrative staff were given a co-ordinating role (Taransay and Hirta Practices), but were of less value in small practices where the intervention was being delivered by fewer people.

“…we started immediately…a list is issued by (administrative staff member named), em to whoever perceived to be the em doctor who sees most of that patient to action it each week, and then it’s, the boxes are ticked and it’s gone back to them to compute.” (Taransay Practice, GP 1 interview)

#### Newsletters

Newsletters, summarising practice performance using data from the tool, were sent to practices at eight weekly intervals. They were circulated around GPs in most practices, but were not formally discussed in any practice participating in the process evaluation (an example is shown in Additional file 2). In practices which immediately implemented DQIP, GPs felt the newsletters were nice to receive to see their targeted ‘high-risk’ prescribing reducing. In two small practices, with immediate implementation, the GPs responsible for the review work found the run charts showing practice progress motivating. Similarly, in two of the practices which initially did not implement the intervention, GPs felt the newsletters were motivating and pushing them into revisiting the tool and review medication.

“Oh yes! Yes we did … yeah we did, em … yeah it kind of pushed us into doing it (reviewing prescribing)…when we got the monthly newsletter.” (Hellisay Practice, GP 2 Interview)

### Informatics tool

In order to be paid, GPs had to use the informatics tool to record review decisions. The tool provided feedback on changes over time in targeted prescribing (run charts), identified patients requiring a review, supported review by summarising relevant clinical information extracted from multiple areas of the GP clinical record, and allowed recording of review decisions which then determined whether and when the patient would be identified as needing review again. Although some practices initially intended to use a print out of identified patients to do reviews, all but one GP carried out the medication reviews online using the tool (see Additional file 3, for example, screenshots). All GPs found the tool intuitive, straightforward and well structured, as the quote below illustrates:

“I liked it — I think we both did — it was, it was easy to use, it was intuitive, you didn’t need a half day tutorial on how to use the blessed thing.” (Hellisay Practice, GP 2 Interview)

The tool’s case finding ability was particularly attractive, especially identifying historical risk factors which GPs felt they were likely to overlook when carrying out a medication review using the same data in their own electronic medical record (e.g. previous peptic ulcer). The GPs also valued the focused data presentation, which only displayed information relevant to the prescribing decision.

“…it’s very straightforward in that you have the on-line tool and it gives you a very structured way of doing it which is, once you get into it much quicker, and other stuff that we do for the sort of GMS and the quality, the National Quality indicator things, em all involve us having to go in and do searches and find people and you know a lot of it’s done on bits of paper and things, so it, it’s done but it’s not, it’s not as easy, it’s, it’s actually made doing this really quite simple and that the tools you know, searches for the patients for you and things which is really good.” (Mingulay Practice, GP 2 Interview)

Although GPs were very positive about the tool, they would prefer a tool which was able to write to their clinical records to prevent double entry of review decisions, for example:

“I think it would be great, the only thing that would be really, really good is if it could talk to Vision [the clinical IT system]…” (Mingulay Practice, GP 1 Interview)

The majority of GPs also expressed a desire for a real-time intervention triggered by an alert when the patient consulted:

“…at the time of the prescription being done rather than retrospectively looking back at it, so that the prescription’s not issued in the first place, might be a good idea.” (Monach Practice, GP1 Interview)

However, a number of GPs preferred a DQIP-like intervention which retrospectively corrected prescribing, because they felt consultations were already heavily time constrained and preferred reviewing outside of clinical consultation to allow sufficient time to adequately review medication.

“Correcting past behaviour … that can make it … it’s a bit of a frustration but I can’t see how it would be otherwise really in the sense that, I don’t know how you fancy an alarm going off every time you sort of try to prescribe something…” (Gighay Practice, GP 1 Interview)

There were no differences in opinion about the informatics tool between professionals in practices which initially responded to the intervention delivered to them and those who did not.

### Performance graphs (run charts)

Within the informatics tool were run charts of change in high-risk prescribing over time where practices could review their progress. These depicted the trend in their targeted high-risk prescribing 2-years pre-DQIP and since they started receiving the DQIP intervention. The run charts in the tool were not perceived to be of value by most GPs due to the relatively small number of patients identified in most practices, although as described above, the same run charts in the newsletters were motivating in some practices.

Although the component parts of the DQIP intervention were important at different stages of recruitment and delivery of the intervention, they also had interactions which were important for effective delivery and implementation. Consistent branding of the DQIP intervention and the financial component ensured GPs were on-board and engaged, the educational component provided clear messages which helped ensure consistency when making prescribing decisions using the tool, and the newsletters provided feedback and motivation to ensure engagement was maintained.

## Discussion

Our findings have shown that all primary components of the intervention were ‘active’, but at different stages. The financial incentive was perceived as most important during recruitment because it acknowledged the additional work required and offered a means of generating extra income. The education including the focus on patient safety reflected in the way the work was branded, motivated the GPs to prioritise this work during initial implementation, and was valued because it provided clear and concise prescribing advice. Notably, many GPs perceived that some or all of the elements of education were useful but not essential, but individuals varied in terms of which elements they valued most. The informatics tool was crucial in case-finding patients, in facilitating efficient medication review decisions, and in implementing the required changes. Many GPs expressed a desire for a real-time alerting element to the informatics as well as support for retrospective review (although it is important to recognise that the majority of the targeted indicators would have triggered an interaction alert at the point of prescribing, consistent with point of care reminders not being a panacea in this area).

Overall, all respondents perceived that some of the components and sub-components were active and synergistic, but there was a variation between participants in which were most valued or perceived to be most active. There was therefore no clearly or consistently identified set of inactive components, and our interpretation is that this suggests that all component parts should be delivered in any further roll-out. However, our prior expectations of how and when different components would be active were not always correct. In particular, financial incentives were perceived by participants as less important than we anticipated, being active in recruitment and initial engagement, but not in promoting sustained delivery to all eligible patients (notwithstanding the caveats described above about how professionals might talk about such incentives in interviews). Although, the run charts and newsletters presented the same data, the run charts providing feedback on progress in the informatics tool were rarely looked at whereas the newsletters were valued in some practices possibly because the latter were accompanied by some individualised interpretation, emphasising that delivery mechanisms matter as well as what is being delivered. This is consistent with the wider audit and feedback literature which emphasises that feedback needs to be optimised to context to maximise effectiveness, although this study was not designed to examine this directly [25].

It is important to note that professional interview data about financial incentives can be difficult to interpret, since some professionals may find it morally ambiguous to say that they require money to improve safety, making it more likely that they say that the incentives did not alter their actions. Nevertheless, some practices had to be repeatedly reminded to invoice for the work done, supporting the belief that in at least some practices, financial incentives played a limited role in mediating effectiveness even though the offer of payment helped to get initial engagement during recruitment.

It is also worth noting that the targeted prescribing topic and intervention were intertwined, in that participants were already mostly persuaded that the targeted prescribing was risky, and their perceptions of the intervention components have to be understood in this context. One implication is that the same intervention components might not be as effective if targeting a prescribing topic that GPs did not perceive as important, or which they felt was less under their control. This is consistent with the findings of a trial of a much simpler feedback intervention in Scotland where five of the targeted measures were similar to DQIP and reduced, but the sixth measure targeting anti-psychotic prescribing in older people was unaffected by the intervention [26].

The normalisation process theory construct of coherence was useful in identifying and describing the components and sub-components of the intervention and when they were useful from the perspective of those who had engaged in some from with the DQIP intervention. For the analysis presented in this paper, we found normalisation process theory to be useful in understanding the nuances associated with collective implementation of the DQIP intervention in general practices where much clinical work is shared.

This study has a number of limitations. The GPs and practices which participated in this study are a sample of approximately a third of the whole trial population and were sampled as outliers at both ends of the distribution of initial success in reducing prescribing, so their views may not be truly representative. Data collection and analysis were carried out by a researcher (AG) who had involvement in the development of the intervention although the researcher with primary responsibility (TD) for the intervention development and the trial had no input into analysis and interpretation of this study. AG did have discussions with BG about the data analysis and interpretation. BG is responsible for the research programme grant, meaning that analysis had limited external scrutiny and challenge.

The analysis in this paper has focused on overall perceptions of when and how different components of the intervention were effective, but the accompanying case study paper (Process evaluation of the data-driven quality improvement in primary are (DQIP) trial: case-study evaluation of an complex intervention to reduce high-risk primary care prescribing. Submitted) qualitatively examining variation between practices in more detail indicates that this plays out variably in different practices, although variation in implementation is also significantly driven by variability in the barriers experienced. The quantitative analysis reported in the second accompanying paper (Process evaluation of the data-driven quality improvement in primary care (DQIP) trial: quantitative examination of representativeness of the trial participants and heterogeneity of impact. Submitted). Found that 30/33 practices had at least some reduction in high-risk prescribing, which is consistent with the finding in this paper that all involved professionals perceived that some or all of the intervention components were active.

In the wider literature, the DQIP intervention is most like the intervention evaluated in the pharmacist-led information technology intervention for medication errors (PINCER) trial [27]. Important differences were that PINCER targeted a broader range of prescribing topics than DQIP, that it was delivered over 12 weeks by pharmacists, and that it was more standardised in terms of how the pharmacists carried out medication reviews. However, the PINCER process evaluation did not explicitly examine the subcomponents of the intervention [28]. A systematic review of main trial evaluations of pharmaceutical and non-pharmaceutical interventions found evaluations of non-pharmaceutical interventions are significantly less likely to list ‘active ingredients’ (those elements of the intervention intended to lead to change in the outcome, [29], and we have not been able to identify any other process evaluations of cluster-randomised trials of multicomponent complex interventions which have examined the ingredients of complex interventions (although such studies are not easy to find because of poor labelling and reporting of process evaluations more generally) [17, 18].

## Conclusions

This paper provides both a detailed description of the DQIP intervention delivered to practices to motivate and facilitate the intended change in how they delivered care to patients. It uses the TIDieR framework to structure intervention description [15] and examines where and when the intervention components were more and less active. We show that all components of the intervention were active but at different stages of recruitment and delivery of the intervention, and that components were perceived to be interdependent and synergistic in their effects. These findings are important for informing wider implementation, since they facilitate reproducibility. More broadly, we believe that trials of complex interventions should at a minimum report the details of their intervention using a framework such as TIDieR to provide a detailed description of the intended active ingredients of the intervention, but should ideally aim to examine whether and when each of these the chosen ingredients is actually active from the perspectives of the intervention targets.

## Additional files

Additional file 1: Educational outreach visit presentation. (DOCX 699 kb)
Additional file 2: Example of 8 weekly newsletter sent 24 weeks after the practice started the intervention where there was little initial change in prescribing. (DOCX 103 kb)
Additional file 3: Screenshots from the DQIP tool. (DOCX 991 kb)

## Abbreviations

AG: Aileen Grant
BG: Bruce Guthrie
DQIP: Data-driven quality improvement in primary care
EOV: Educational outreach visit
GP: General practitioner
NICE: National Institute of Clinical Excellence
NPT: Normalisation process theory
NSAIDs: Non-steroidal anti-inflammatory drugs
PINCER: A Pharmacist-led Information technology Intervention for Medication Errors
TD: Tobias Dreischulte
TIDieR: Template for intervention description and replication
