# Supplementary figures and images for: Process evaluation of the data-driven quality improvement in primary care (DQIP) trial: active and less active ingredients of a multi-component complex intervention to reduce high-risk primary care prescribing
Source: Implement Sci. 2017 Jan 7;12:4. doi: 10.1186/s13012-016-0531-2 (PMC5219764; doi:10.1186/s13012-016-0531-2)

1. **Educational outreach presentation**

| 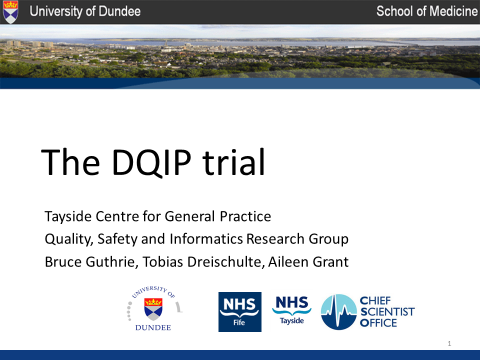 | 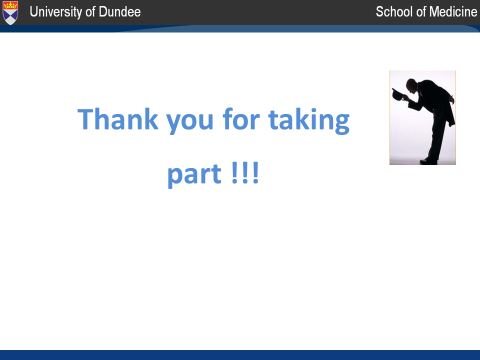 |
| --- | --- |
| 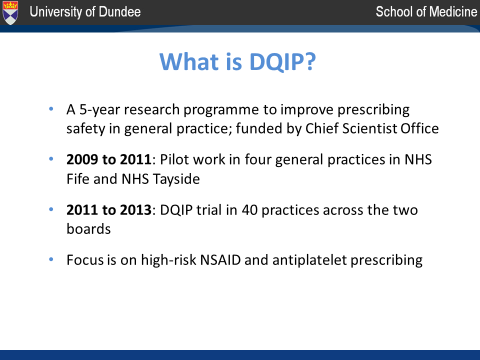 | 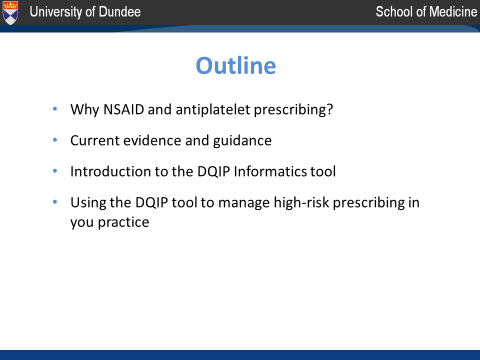 |
| 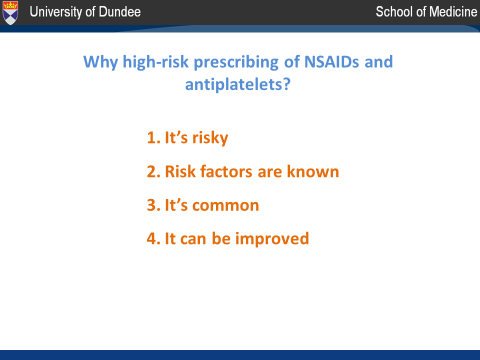 | 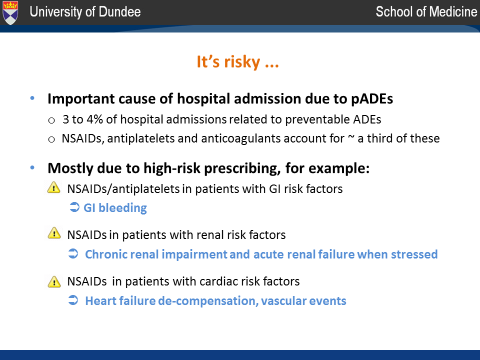 |
| 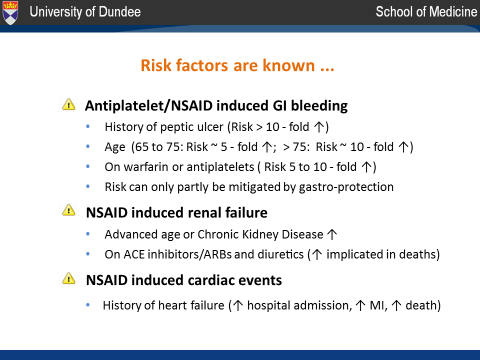 | 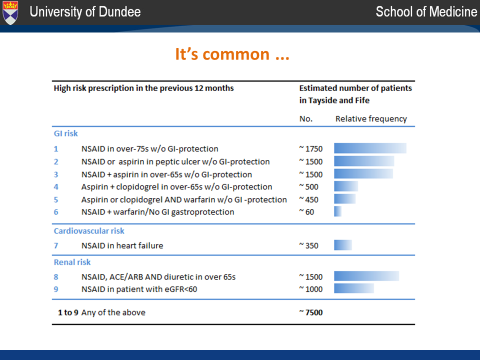 |
| 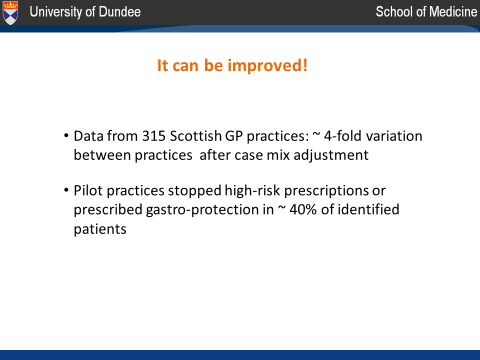 | 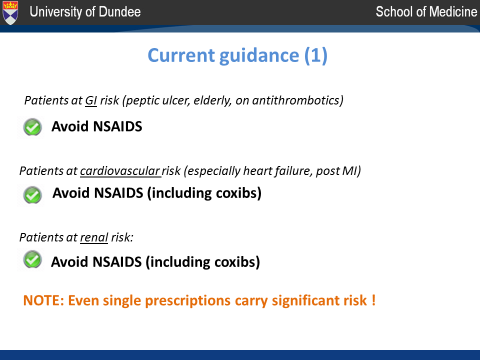 |
| 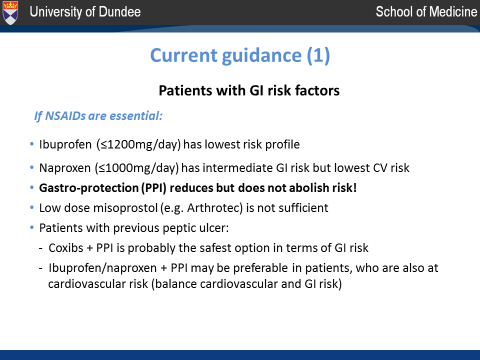 | 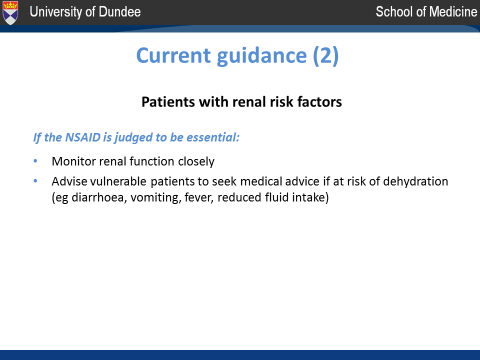 |
| 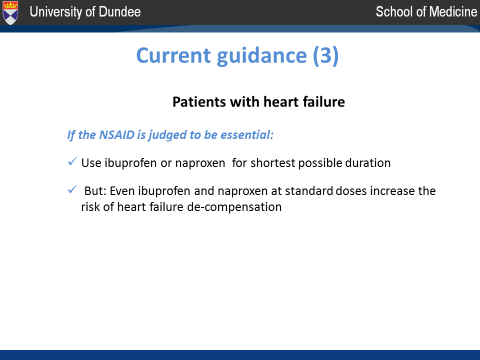 | 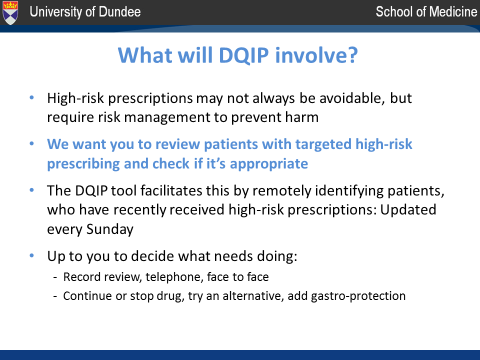 |
| 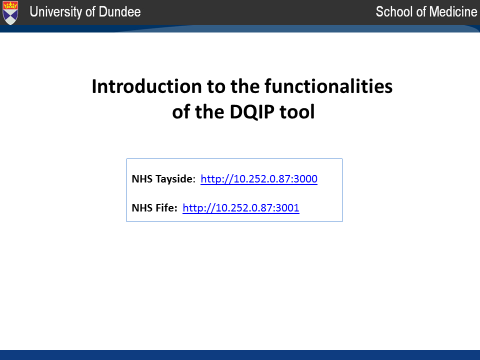 | 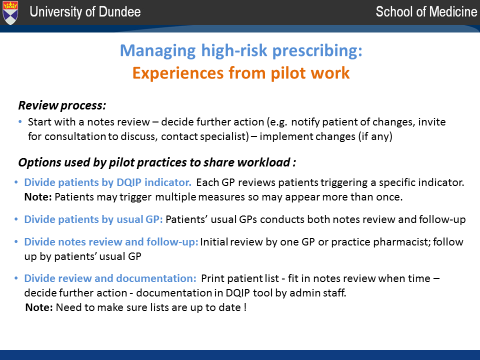 |


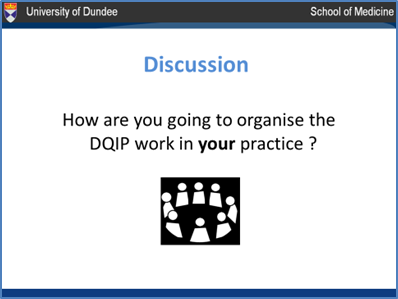

Supplement: Additional file 1: — Educational outreach visit presentation. (DOCX 699 kb) [file 13012_2016_531_MOESM1_ESM.docx]
