# Supplementary material for: Process evaluation of the data-driven quality improvement in primary care (DQIP) trial: active and less active ingredients of a multi-component complex intervention to reduce high-risk primary care prescribing
Source: Implement Sci. 2017 Jan 7;12:4. doi: 10.1186/s13012-016-0531-2 (PMC5219764; doi:10.1186/s13012-016-0531-2)
Supplement: Additional file 2: — Example of 8 weekly newsletter sent 24 weeks after the practice started the intervention where there was little initial change in prescribing. (DOCX 103 kb) [file 13012_2016_531_MOESM2_ESM.docx]

1. **Example of 8 weekly newsletter sent 24 weeks after the practice started the intervention where there was little initial change in prescribing**


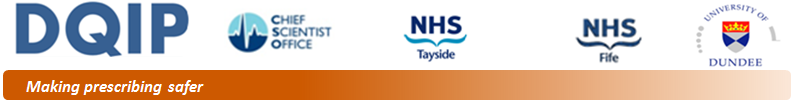


**Dear GP/PM**,

Six months have now passed since your practice has started the DQIP programme, with 6 months remaining. We are writing to you today in order to give you an update on your progress with the DQIP work.

***Trends in high-risk prescribing***

The run chart below (screenshot from the DQIP tool) shows that the total numbers of patients affected by any high-risk NSAID or antiplatelet prescribing have dropped to some extent since the last update 8 weeks ago, but the overall level of high-risk prescribing has remained similar to that seen before you’ve started DQIP. Figure 2 shows an update of the run chart of a practice who have successfully minimised their high-risk prescribing over 6 months.

| **Figure 1:** Run chart for your practice | | | **Figure 2:** Run chart for a practice with comparable numbers of patients affected at DQIP start |
| --- | --- | --- | --- |
| 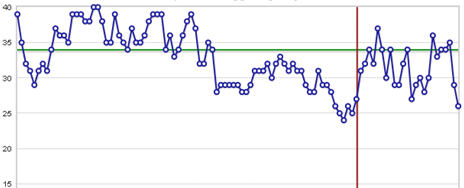 | | | 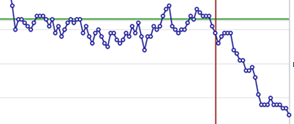 |
|  |  | DQIP start date | |
|  |  |  |  |
|  |  | Mean number of patients before DQIP start | |

We know it is challenging to reduce high-risk prescribing at practice level and maintain such reductions over time, because it requires not only stopping high-risk prescriptions in patients who have been flagged up by the DQIP tool, but also avoiding new high-risk prescriptions in patients with risk factors. This is especially the case when not all prescribers in the practice are involved in conducting the DQIP reviews. Where it is not possible to involve all prescribers in the review work, alerting colleagues when high-risk prescriptions have been issued may be crucial to increase awareness of future high-risk situations, in which NSAIDs and antiplatelets should be avoided or risk mitigating strategies implemented (if avoidance is not possible).

***Can we help?***

Please let us know if there is anything we can do to help (including a further practice visit if you think this would be helpful). You can contact us via email at [dqip@dundee.ac.uk](mailto:dqip@dundee.ac.uk) or by phone under 01382-420000.

***Medication reviews***

The practice has reviewed a total of 42 patients since the beginning of the DQIP trial corresponding to a payment of £630. There are currently 8 patients in your practice, who have yet to be reviewed. Please note that because we are measuring high-risk prescribing in the last 8 weeks, the *full* impact of any medication changes made may take a few weeks to show up in the run chart.

Kind regards,

***The DQIP team***
