# Supplementary material for: Process evaluation of the data-driven quality improvement in primary care (DQIP) trial: active and less active ingredients of a multi-component complex intervention to reduce high-risk primary care prescribing
Source: Implement Sci. 2017 Jan 7;12:4. doi: 10.1186/s13012-016-0531-2 (PMC5219764; doi:10.1186/s13012-016-0531-2)

**Screenshots from the DQIP tool**

The DQIP tool consisted of five webpages, and screenshots of each page are shown below (all data is fictional).

Figure A1: Overview page (summarises data for each measure and the primary outcome composite measure including a thumbnail of change over time, and numerical summaries of the numbers needing review)


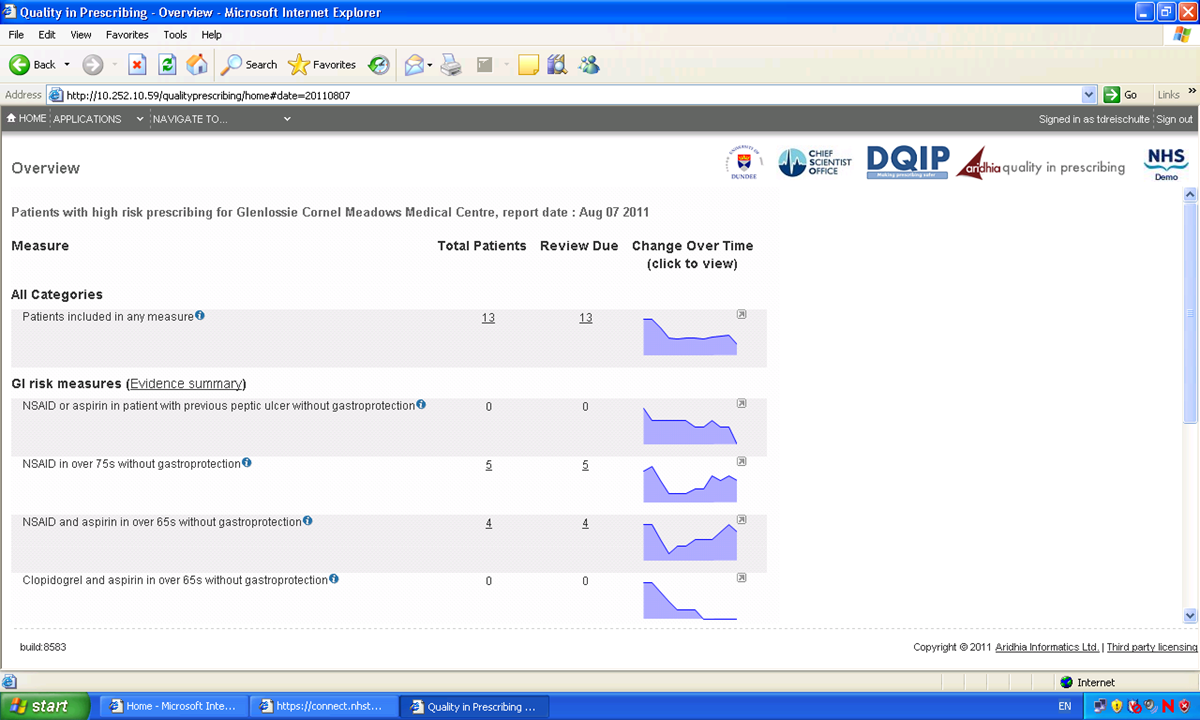


Figure A2: Change over time graph (accessed by clicking on the thumbnail on the overview page; for each indicator, shows how the number of patients with a particular type of prescribing has changed over time in relation to the pre-intervention average and when the practice started the intervention)


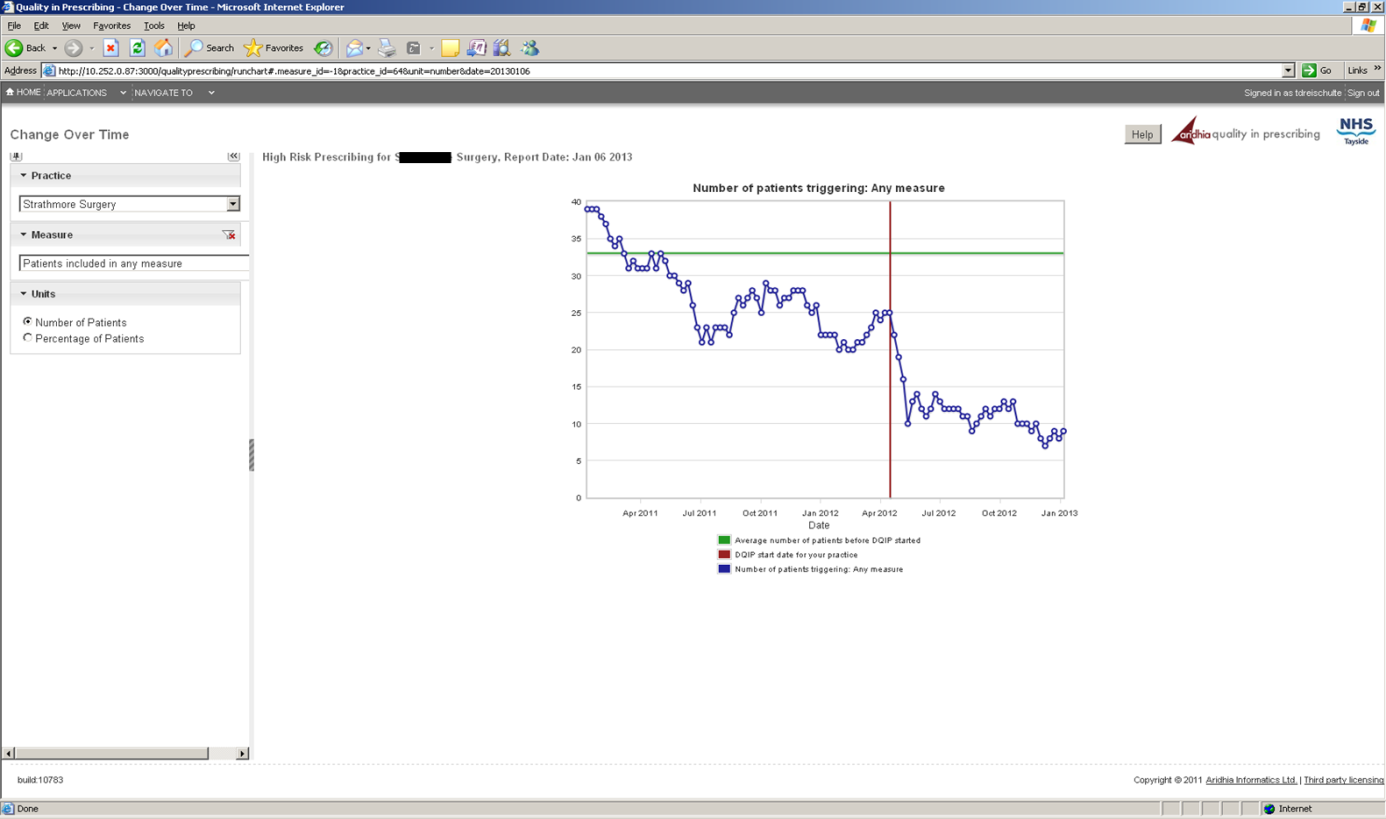


Figure A3: Patient list (accessed by clicking on one of the numbers on the overview page; lists patients needing review or already reviewed)


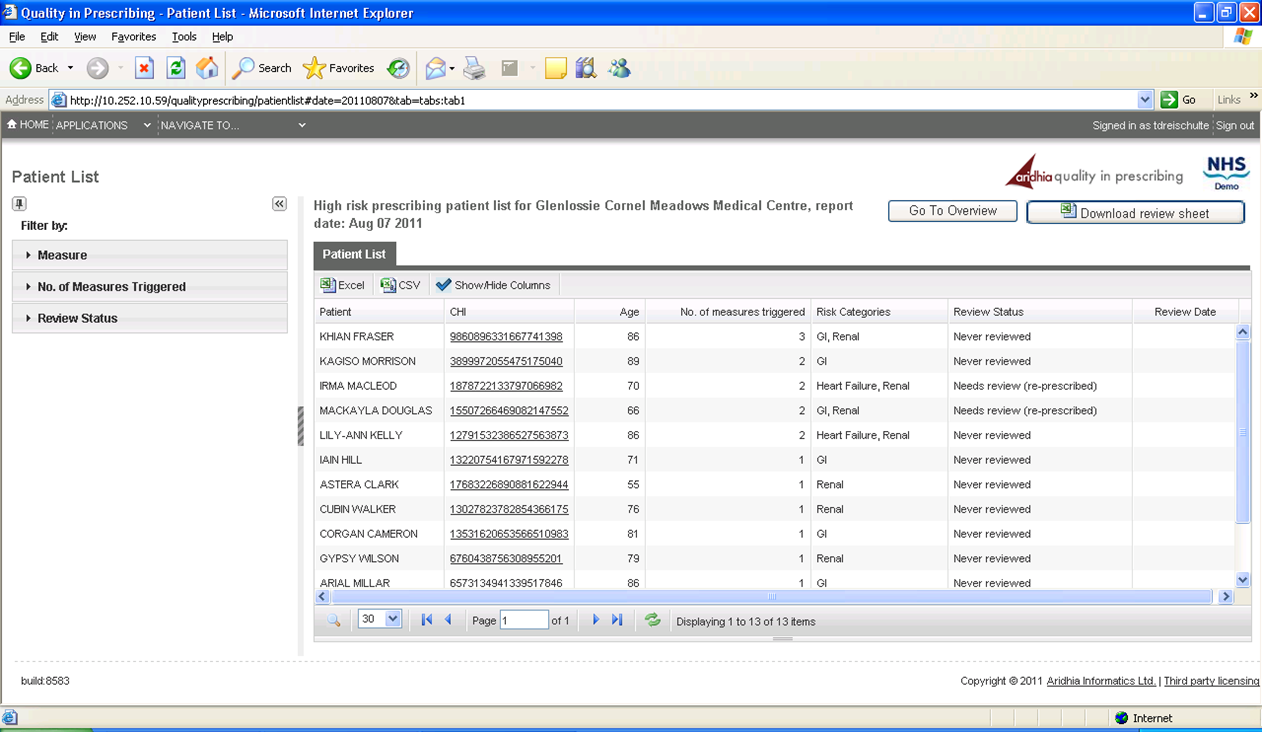


Figure A4: Prescribing detail page (accessed by clicking on a patient on the patient list page; summarises the patient characteristics which makes them vulnerable to targeted prescribing, and their recent relevant prescribing history; hovering over data points creates pop-ups with more details; allows the reviewer to record what they intend to do about identified drugs).


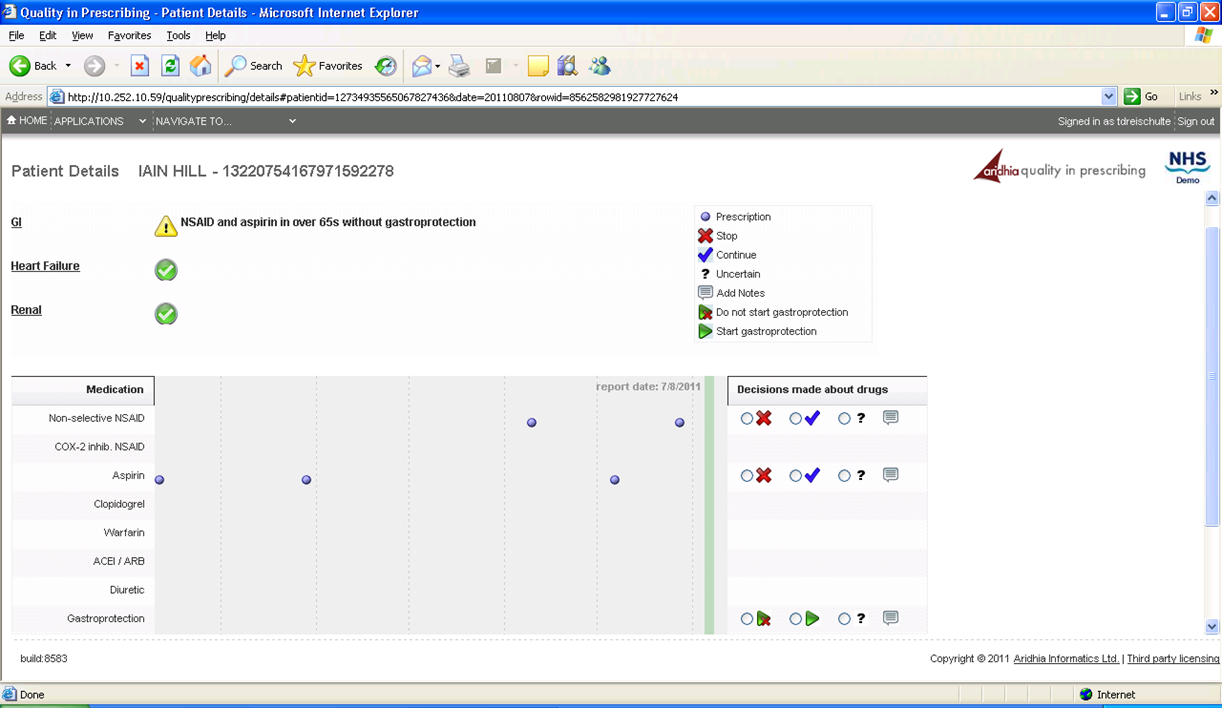


Figure A5: Recording review decisions page (accessed when exiting from the prescribing details page; records what action the reviewer judges necessary).


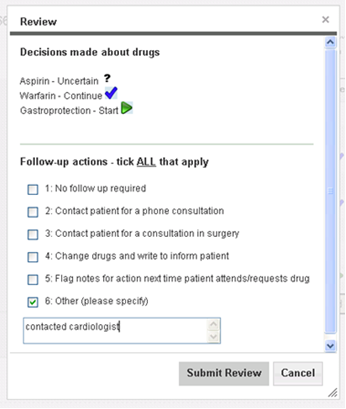

Supplement: Additional file 3: — Screenshots from the DQIP tool. (DOCX 991 kb) [file 13012_2016_531_MOESM3_ESM.docx]
